# Clinical outcomes of vital pulp therapy versus root canal treatment in immature permanent teeth with pulpal involvement: a meta-analysis

**DOI:** 10.3389/fped.2026.1754580

**Published:** 2026-05-20

**Authors:** Siji Gu, Yuyun Yang

**Affiliations:** 1 Department of Pediatric Dentistry, Shaoxing Stomatological Hospital, Shaoxing, Zhejiang, China; 2 Department of Preventive Dentistry, Shaoxing Stomatological Hospital, Shaoxing, Zhejiang, China

**Keywords:** apexogenesis, immature permanent teeth, meta-analysis, root canal treatment, vital pulp therapy

## Abstract

**Objectives:**

This meta-analysis evaluated whether vital pulp therapy (VPT) provides more favorable outcomes compared with root canal treatment (RCT) in immature permanent teeth with pulpal involvement. Given the pivotal role of pulp vitality in apexogenesis, the study aimed to determine whether biologically conservative interventions offer greater clinical and radiographic success than pulpectomy-based therapies.

**Methods:**

A comprehensive search of PubMed, Embase, Cochrane Library, and Web of Science was conducted from inception to the final search date. Eligible studies included randomized controlled trials and comparative clinical cohorts evaluating VPT (partial, cervical, or complete pulpotomy; direct/indirect capping) vs. RCT in immature permanent teeth. The primary endpoint was composite clinical–radiographic success at the longest follow-up. Data were extracted at the tooth level and pooled using random-effects models to estimate odds ratios (ORs) with 95% confidence intervals (CIs). Prespecified subgroup analyses examined the effects of tooth maturity, VPT modality, biomaterial, etiology, and follow-up duration. Meta-regression explored determinants of treatment success, and trial sequential analysis (TSA) was performed to assess the sufficiency of cumulative evidence.

**Results:**

Eight studies were included qualitatively, and five contributed tooth-level data (*n* = 482 teeth). VPT demonstrated significantly higher odds of overall success compared with RCT (pooled OR = 1.37, 95% CI: 1.06–1.78, *p* = 0.027), with negligible heterogeneity (I^2^ = 0%). Subgroup analyses showed marked benefit in immature teeth (OR = 3.01, 95% CI 1.43–6.33, *p* = 0.004), complete/coronal pulpotomy (OR = 2.47, 95% CI 1.11–5.51, *p* = 0.026), and follow-up ≥24 months (OR = 2.03, 95% CI 1.21–3.41, *p* = 0.002). Bioceramic-based VPT achieved near-complete success in randomized trials (PMTA/CEM: 98/98 teeth), whereas RCT outcomes plateaued at 79%–98%. Meta-regression identified tooth immaturity, full pulpotomy, and longer follow-up as positive prognostic factors. TSA showed that the cumulative Z-curve crossed the conventional threshold but not the monitoring boundary or required information size, indicating a favorable direction of effect but insufficient evidence for a definitive TSA-adjusted conclusion.

**Conclusions:**

VPT was associated with favorable long-term outcomes compared with RCT in immature permanent teeth. However, because the cumulative evidence did not reach the trial sequential monitoring boundary or the required information size, the certainty of this apparent advantage remains limited. Preservation of pulp vitality may support apexogenesis and structural development, but larger comparative studies are still needed before definitive superiority can be concluded.

## Introduction

Dental caries remains the most widespread non-communicable disease globally and constitutes a primary driver of pulpal pathology in children and adolescents (1). As bacterial acids penetrate the dentin–pulp complex, they initiate localized inflammation, alter nociceptive signaling, and compromise pulpal homeostasis (2–4). Without timely management, these processes extend deeper into the pulp, often provoking clinical symptoms such as prolonged thermal sensitivity or spontaneous pain, historically interpreted as hallmarks of irreversible pulpitis (5). Root canal treatment (RCT) subsequently emerged as the conventional solution, built upon the assumption that once inflammation reaches a certain threshold, the pulp becomes incapable of healing. This mindset, though effective in retaining teeth, reflects a conceptual framework in which the pulp is viewed more as a liability than as a biologically active tissue.

RCT offers predictable microbial control and long-term survival when executed to high technical standards (6, 7). Yet its biological consequences are profound: complete pulpectomy eliminates vascular supply, immune surveillance, and intrinsic regenerative capacity (8). It also compromises the biomechanical function of dentin. Dehydration of tubules, loss of intradental pressure, and extensive instrumentation weaken the remaining tooth structure (9). These effects disproportionately affect young patients, whose permanent teeth exhibit wide canals, thin radicular walls, and incomplete root maturation. In addition, RCT entails considerable chair time, cost, and technical demand, which can limit accessibility and exacerbate inequalities in oral healthcare (10–12). Across many health systems, extraction remains the default for advanced pulpitis precisely because RCT is often unaffordable or unavailable. Such outcomes underscore an unmet need for treatment modalities that are biologically respectful, technically feasible, and economically sustainable.

Vital pulp therapy (VPT) addresses this need by re-framing pulpal disease as a potentially reversible, regionally distributed process rather than a binary pathological state (13). Instead of removing the entire pulp, VPT selectively excises infected or irreversibly inflamed tissue while retaining the remaining vital pulp. This approach leverages the pulp's capacity for repair and regeneration, a capacity increasingly recognized through modern histopathology. Even in symptomatic cases, inflammation may be confined to the coronal pulp, while deeper regions retain an intact microvascular network and functional odontoblast populations (14, 15). Techniques such as direct or indirect pulp capping, partial pulpotomy, and full pulpotomy aim to preserve this biological substrate. The emergence of calcium silicate–based biomaterials has further accelerated the adoption of VPT. These bioceramic materials generate alkaline microenvironments, control bacterial contamination, release bioactive ions, and stimulate dentin bridge formation, supporting an environment conducive to healing (16). Collectively, these advances have challenged long-standing assumptions that irreversible pulpitis must inevitably lead to pulpectomy.

The biological argument for VPT is especially compelling in immature permanent teeth. Unlike mature dentition, immature roots remain structurally and physiologically dependent on the pulp for continued dentin deposition and apical closure (17). Preservation of the pulp allows root elongation, thickening of dentinal walls, and development of long-term structural resilience. In contrast, premature pulpectomy interrupts these processes, yielding teeth that are short-rooted, fragile, and prone to fracture. Although apexification and regenerative endodontic procedures attempt to compensate for the loss of pulpal function, both require prolonged intervention, introduce technique sensitivity, and do not fully restore the native physiological environment (18). A biologically intact pulp, when maintained, is therefore not merely a convenience-it is essential to the developmental trajectory of the tooth. From this perspective, immature permanent teeth are not “small adult teeth”; they are actively evolving tissues that demand distinct therapeutic considerations.

Immature permanent teeth possess several biological characteristics that favor vital pulp preservation. The presence of wide apical foramina and a rich vascular supply facilitates immune defense, nutrient exchange, and reparative dentinogenesis within the pulp tissue (19). When inflammation remains confined to the superficial coronal pulp, partial pulpotomy and other forms of vital pulp therapy can maintain pulp vitality and allow continued root development through apexogenesis (20). Clinical guidelines and expert reviews therefore recommend pulpotomy as a first-line treatment in many cases involving immature permanent teeth with limited pulpal inflammation (19). Preservation of the vital pulp not only supports physiological root maturation but also enhances long-term structural stability compared with conventional root canal treatment, which interrupts the natural developmental process of the tooth (21).

Despite mounting biological evidence, clinical management remains inconsistent. Substantial heterogeneity exists in how dentists approach deep caries, pulp exposure, and suspected pulpitis. Some clinicians adopt total caries removal and proceed directly to RCT to avoid uncertainty; others apply selective excavation or staged interventions (22). These differences are magnified by diagnostic ambiguity. Clinical symptoms alone lack specificity, and thermal or electrical testing assesses neural response rather than tissue vitality (23). Radiographs provide structural but not inflammatory insight. As a result, many immature teeth are overtreated due to fear of failure, while others are undertreated due to optimism regarding spontaneous recovery. This diagnostic gap is not trivial. For the growing tooth, inappropriate RCT sacrifices the very biological machinery required for maturation, while delayed treatment risks progression to necrosis and apical disease. The challenge is therefore not merely technical-it is conceptual: choosing a therapy aligned with pulp biology rather than clinical heuristics.

Amid this uncertainty, one question remains insufficiently answered: How do the clinical outcomes of VPT compare with those of RCT specifically in immature permanent teeth with pulpal involvement? Most comparative evidence arises from mature dentition, where structural conditions and treatment goals differ fundamentally. Recent studies have also suggested that vital pulp therapy may be a feasible treatment option in mature permanent teeth with pulpitis, although reported success rates are generally lower than those observed in immature teeth (24). Pediatric epidemiological studies describe treatment distributions but rarely quantify outcomes against RCT. Trauma-focused cohorts introduce confounding through vascular injury or luxation. Systematic reviews often pool immature and mature teeth, obscuring age-dependent differences in healing potential. Consequently, clinicians lack a unified evidence base to guide treatment decisions for immature teeth-a population in which the consequences of overtreatment or undertreatment can persist for decades. Therefore, this meta-analysis aims to compare the clinical outcomes of VPT and RCT in immature permanent teeth with pulpal involvement. By isolating this specific developmental context, we seek to clarify whether VPT provides non-inferior or superior outcomes relative to RCT and whether a biological preservation strategy should be prioritized in young permanent dentition. This analysis is designed not simply to revisit historical debate, but to inform evidence-based practice, ensure developmental integrity, and align contemporary clinical decision-making with the principles of regenerative dentistry and lifelong oral health.

## Methods

### Study design and eligibility criteria

**Figure 1:**
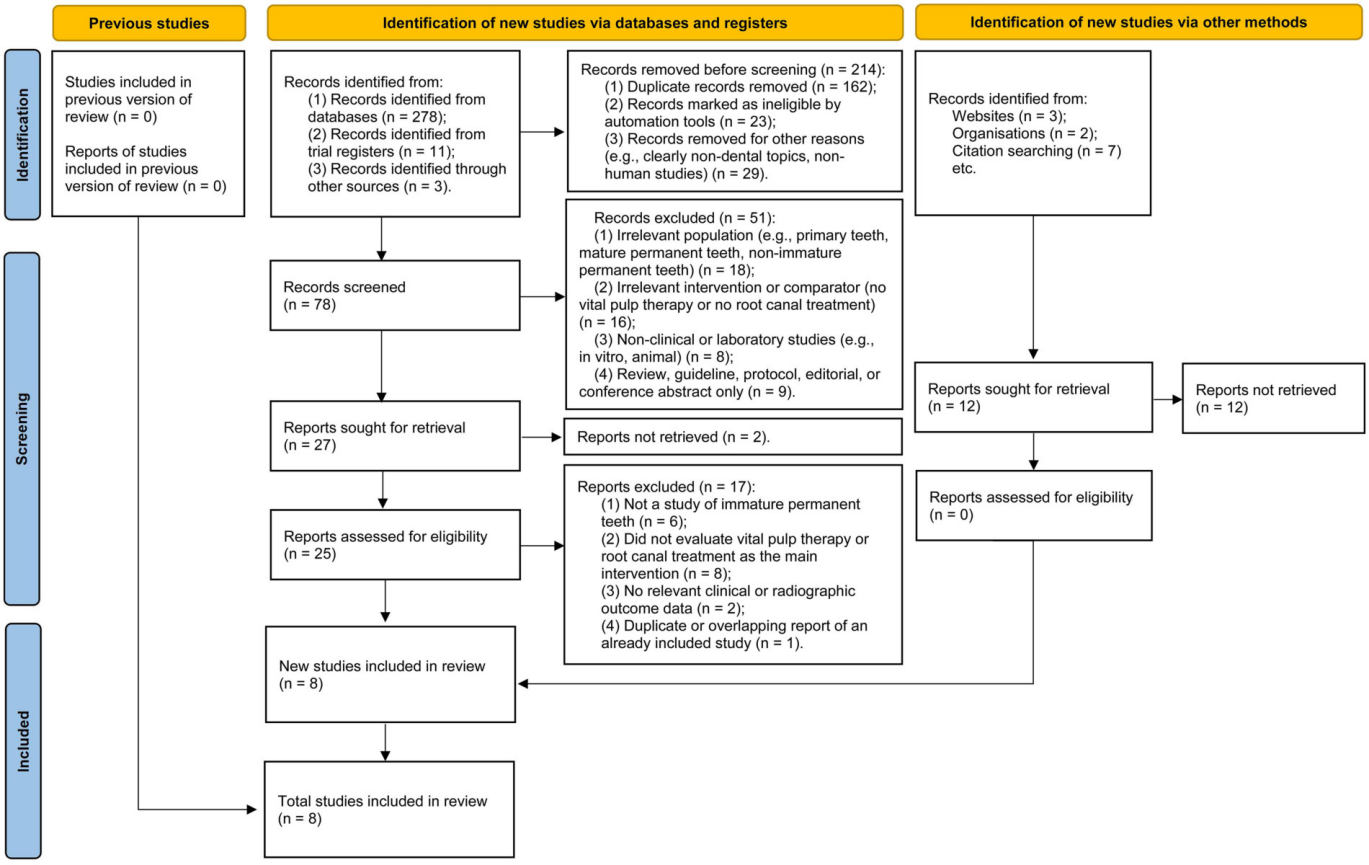
PRISMA 2020 flow diagram of study selection. This figure summarizes the identification, screening, eligibility, and inclusion process used in this review. A total of 292 records were identified from four electronic databases and supplementary sources. After removal of duplicates and non-relevant entries, 78 articles underwent title/abstract screening, with 27 full-text reports assessed for eligibility. Eight studies met the inclusion criteria for qualitative synthesis, and five provided extractable tooth-level outcome data suitable for quantitative meta-analysis. Reasons for study exclusion at each stage (e.g., wrong population, absence of VPT/RCT comparison, no extractable outcomes, overlapping cohorts) are listed in the diagram in accordance with PRISMA 2020 standards.

This study was designed as a systematic review and meta-analysis comparing the clinical outcomes of VPT and RCT in immature permanent teeth with pulpal involvement. The review was conducted according to the Preferred Reporting Items for Systematic Reviews and Meta-Analyses (PRISMA) 2020 guidelines (Figure 1) (25). Ethical approval was not required because the analysis synthesized data derived exclusively from previously published studies without direct patient contact. The target population comprised patients presenting with immature permanent teeth, characterized radiographically or clinically by incomplete root formation, open apices, or active apexogenesis. Pulpal involvement was defined as a pathological or iatrogenic exposure of the pulp secondary to dental caries, trauma, restorative procedures, or mixed etiology, provided that pulpal pathology was clinically or radiographically evident. Studies focusing on mature teeth or primary dentition were excluded, as treatment objectives and biological healing capacities differ fundamentally in these populations.

Eligible studies met the following inclusion criteria: (1) the intervention involved a form of vital pulp therapy (VPT) aimed at preserving viable pulp tissue and enabling continued root development, including direct pulp capping, indirect pulp capping, partial pulpotomy, full pulpotomy, or coronal pulpotomy; (2) the comparator consisted of conventional root canal treatment (RCT), defined as mechanical and chemical debridement of the entire pulp space followed by obturation using gutta-percha or alternative materials, including single- or multi-visit RCT protocols as well as apexification or apical barrier procedures for immature roots; (3) no restrictions were imposed on biomaterials used for VPT, allowing inclusion of calcium silicate–based bioceramics (e.g., mineral trioxide aggregate, Biodentine), calcium hydroxide, resin-modified glass ionomers, bioceramic putties, or composite restorations; and (4) regenerative endodontic procedures were considered eligible only when they were explicitly designed as comparative arms to VPT and reported independent outcome measures.

Studies were excluded according to the following criteria: (1) the primary intervention involved complete pulp removal procedures (e.g., pulpectomy) without a VPT comparator; (2) the study did not allow extraction of relevant clinical outcome data; or (3) the intervention was not consistent with the biological objective of vital pulp preservation.

To be considered eligible, studies were required to provide extractable clinical outcome data. The primary outcome was treatment success, defined as a composite measure integrating both clinical and radiographic success. Clinical success was characterized by the absence of spontaneous pain, swelling, percussion sensitivity, sinus tract formation, pathological mobility, or signs of acute exacerbation. Radiographic success included absence of periapical radiolucency and evidence of continued root development, including elongation of the root, thickening of radicular dentinal walls, or apical closure. Secondary outcomes, such as clinical success alone, radiographic success alone, tooth survival, or postoperative complications, were also extracted when available. Studies reporting surrogate or laboratory endpoints, such as biomaterial microstructure or histological findings without clinical follow-up, were excluded.

Original studies with parallel-group comparative designs—including randomized controlled trials, prospective cohorts, retrospective controlled cohorts, and case-control studies—were eligible for inclusion. Case series without controls, cross-sectional surveys with no direct comparison, review articles, conference abstracts, *in vitro* or *ex vivo* studies, and animal experiments were excluded because they do not support effect size calculation or clinically meaningful inference. No restrictions were imposed on patient age, clinical setting, operator experience, or biomaterial selection. When studies reported multiple follow-up intervals, the longest available follow-up of at least 12 months was prioritized to reflect long-term biological healing and functional tooth maturation. For studies that presented overlapping cohorts or duplicate data sources, the most comprehensive or most recent dataset was selected to avoid redundancy and ensure methodological integrity.

### Search strategy

A comprehensive electronic search strategy was developed to identify all relevant clinical studies comparing VPT with RCT in immature permanent teeth with pulpal involvement. The search was conducted in four major bibliographic databases (PubMed, Embase, the Cochrane Library, and Web of Science) from database inception to November 2025, when the final search update was performed. For each database, combinations of controlled vocabulary terms and free-text keywords were used to capture four core conceptual domains: the intervention of interest (VPT, including pulpotomy and pulp capping), the comparator (RCT or endodontic therapy), the target population (immature permanent teeth, open apex, apexogenesis), and the pathological condition (pulpitis, irreversible pulpitis, pulpal involvement). In PubMed and Embase, Medical Subject Headings (MeSH) and Emtree terms were combined with title/abstract terms using Boolean operators and truncation where appropriate, while in the Cochrane Library and Web of Science, an expanded keyword-based strategy was applied at the title, abstract, and topic levels. The full database-specific search strings, including all operators and subject headings, are presented in Table 1 to facilitate reproducibility.

**Table 1:** Search strategy.

Database	Search strategy	Subject headings/keywords
PubMed	((“Vital Pulp Therapy”[Mesh] OR “vital pulp therapy”[tiab] OR pulpotomy[tiab] OR “partial pulpotomy”[tiab] OR “full pulpotomy”[tiab] OR “coronal pulpotomy”[tiab] OR “pulp capping”[tiab] OR “direct pulp capping”[tiab] OR “indirect pulp capping”[tiab]) AND (“Root Canal Therapy”[Mesh] OR “root canal treatment”[tiab] OR “root canal therapy”[tiab] OR “endodontic therapy”[tiab] OR “endodontic treatment”[tiab]) AND (“Tooth, Permanent”[Mesh] OR “immature permanent teeth”[tiab] OR “immature permanent tooth”[tiab] OR “open apex”[tiab] OR “apexogenesis”[tiab]) AND (“Pulpitis”[Mesh] OR pulpitis[tiab] OR “pulpal involvement”[tiab] OR “irreversible pulpitis”[tiab] OR “pulp necrosis”[tiab]))	MeSH: Vital Pulp Therapy; Root Canal Therapy; Tooth, Permanent; Pulpitis.
		Keywords: VPT, pulpotomy, apexogenesis, immature permanent teeth, open apex, endodontic therapy, root canal therapy.
Am	(“vital pulp therapy”/exp OR “pulpotomy”/exp OR “vital pulp therapy”:ti,ab OR pulpotomy:ti,ab OR “partial pulpotomy”:ti,ab OR “coronal pulpotomy”:ti,ab OR “pulp capping”:ti,ab OR “direct pulp capping”:ti,ab OR “indirect pulp capping”:ti,ab) AND (‘root canal therapy’/exp OR “root canal treatment”:ti,ab OR “endodontic therapy”:ti,ab OR “endodontic treatment”:ti,ab) AND (‘permanent tooth’/exp OR “immature permanent tooth”:ti,ab OR “immature permanent teeth”:ti,ab OR “open apex”:ti,ab OR apexogenesis:ti,ab) AND (‘pulp inflammation’/exp OR ‘pulpitis’/exp OR pulpitis:ti,ab OR “irreversible pulpitis”:ti,ab OR “pulpal involvement”:ti,ab)	Emtree: vital pulp therapy; pulpotomy; root canal therapy; permanent tooth; pulp inflammation.
		Keywords: immature permanent teeth, apexogenesis, endodontic treatment.
Cochrane Library	(“vital pulp therapy” OR pulpotomy OR “partial pulpotomy” OR “pulp capping” OR “direct pulp capping” OR “indirect pulp capping”) AND (“root canal treatment” OR “root canal therapy” OR “endodontic therapy”) AND (“immature permanent teeth” OR “immature permanent tooth” OR “open apex” OR apexogenesis) AND (pulpitis OR “irreversible pulpitis” OR “pulpal involvement”)	Clinical dental terms and procedural keywords.
Web of Science	TS = (“vital pulp therapy” OR pulpotomy OR “partial pulpotomy” OR “pulp capping” OR “direct pulp capping” OR “indirect pulp capping”) AND TS = (“root canal treatment” OR “root canal therapy” OR “endodontic therapy” OR “endodontic treatment”) AND TS = (“immature permanent teeth” OR “immature permanent tooth” OR “open apex” OR apexogenesis) AND TS = (pulpitis OR “irreversible pulpitis” OR “pulpal involvement”)	Topic keyword set aligned to VPT vs. RCT comparison.

Searches were conducted in PubMed, Embase, the Cochrane Library, and Web of Science from database inception to the final search date. Both controlled vocabulary (e.g., MeSH in PubMed, Emtree in Embase) and free-text terms (title/abstract keywords) were used to maximize sensitivity. Search strings were iteratively refined to capture variations of vital pulp therapy, including pulpotomy (partial/full/coronal) and pulp capping (direct/indirect), which represent standard modalities of VPT in immature permanent teeth. Terms related to the control intervention, including root canal treatment/therapy and endodontic treatment, were incorporated to ensure proper comparator identification. Expressions describing the target population, such as immature permanent teeth, open apex, and apexogenesis, were included because they reflect common terminology in pediatric dentistry and endodontic practice. Clinical diagnostic descriptors (e.g., pulpitis, irreversible pulpitis, pulpal involvement) were added to ensure that studies addressed actual pulpal pathology and not only preventive or restorative procedures. No language or publication date restrictions were applied. Manual reference list screening, forward citation searching, and review of relevant reviews and clinical guidelines were performed to identify additional eligible studies. Duplicates were removed before screening. All retrieved references were screened independently by two reviewers following PRISMA 2020 guidelines.

The initial search strategy was drafted by one reviewer with expertise in endodontics and evidence synthesis and was iteratively refined through pilot searches to optimize both sensitivity and specificity. No restrictions were placed on language, year of publication, or country, and non-English articles were translated when necessary to avoid language bias. In addition to the core database searches, supplementary strategies were employed to minimize the risk of missing eligible studies. These included manual screening of the reference lists of all included articles and relevant reviews, forward citation tracking of key studies using Web of Science, and targeted searches of professional dental organization websites and grey literature sources. Trial registers and other non-indexed sources were also screened where applicable.

### Study selection

All records retrieved from the electronic searches were first exported into reference management software (EndNote X9, Clarivate Analytics) for de-duplication. Titles and abstracts were then screened independently by two calibrated reviewers using pre-specified eligibility criteria based on the PICO framework. Potentially relevant articles and any records for which eligibility could not be confidently determined from the title and abstract were retained for full-text review.

Full-text articles were then obtained and assessed in detail by the same two reviewers, working independently and in a blinded manner with respect to each other's decisions. Each full-text report was evaluated against all inclusion and exclusion criteria, with particular attention to confirmation of immature root status, explicit description of VPT and RCT as the main interventions, and availability of extractable clinical and/or radiographic outcome data. Reasons for exclusion at the full-text stage were recorded systematically to ensure transparency. Any disagreements in study selection at either screening stage were resolved through discussion and, when necessary, arbitration by a third senior reviewer.

### Data extraction

Data extraction was performed independently by two reviewers using a standardized, pilot-tested data collection form designed specifically for this review. For each included study, the following information was recorded: first author's name, year of publication, country or region, study design (randomized controlled trial, prospective cohort, retrospective cohort, or case–control), sample size, patient age or age range, tooth type and location when available, etiology of pulpal involvement (caries, trauma, or mixed), and diagnostic criteria used to define pulp status. Detailed information on the intervention and comparator protocols was also extracted, including the specific type of VPT (direct or indirect pulp capping, partial pulpotomy, full/coronal pulpotomy), biomaterials employed (e.g., calcium silicate–based cements such as mineral trioxide aggregate or Biodentine, calcium hydroxide, or other liners), operative steps, and follow-up regimen. For the RCT group, data were collected on treatment protocol (single- vs. multi-visit), use of apexification or apical barrier techniques, irrigants and intracanal medicaments, obturation materials, and restoration type.

Outcome data were extracted at the tooth level whenever possible. The primary outcome was overall treatment success, defined as the combined presence of clinical and radiographic success according to each study's criteria; where definitions differed slightly across studies, the most stringent composite definition (symptom-free tooth with no clinical signs of failure and no radiographic evidence of periapical disease) was adopted. When reported, separate data for clinical success, radiographic success, continued root development (apical closure, root lengthening, or dentinal wall thickening), tooth survival, and adverse events or reinterventions were also collected as secondary endpoints. For dichotomous outcomes, the numbers of successful and failed teeth in each treatment arm were extracted directly or reconstructed from percentages and total counts. In multi-arm studies comparing more than one VPT modality with a single RCT group, data were split appropriately to avoid double-counting the control group in the meta-analysis. When essential numerical data were unclear or incompletely reported in the original article, attempts were made to contact the corresponding authors for clarification. Any discrepancies between the two reviewers in data extraction were resolved by cross-checking the source articles and reaching consensus, with referral to a third reviewer when necessary.

### Risk of bias assessment

Risk of bias was evaluated using study design–appropriate standardized frameworks to ensure consistent appraisal across randomized and non-randomized evidence. For randomized controlled trials, the revised Cochrane Risk of Bias tool (RoB 2) was employed to assess potential bias in five core domains: adequacy of the randomization process; deviations from intended interventions; completeness of outcome data; reliability of outcome measurement; and selective reporting of results. For non-randomized comparative studies, the Risk Of Bias In Non-randomized Studies of Interventions (ROBINS-I) tool was applied, which estimates bias relative to a hypothetical ideal randomized trial and examines confounding, participant selection, intervention classification, deviations from intended treatment, missing data, outcome ascertainment, and selective reporting. Risk assessments were independently conducted by two trained reviewers, with disagreements resolved through discussion and, when necessary, arbitration by a senior reviewer. Overall risk-of-bias judgments were not used as exclusion criteria; rather, they informed prespecified sensitivity and subgroup analyses to evaluate whether pooled effect estimates were influenced by underlying methodological limitations.

### Outcomes

The primary outcome of interest was overall treatment success, defined as the concurrent achievement of clinical and radiographic success at the final or longest available follow-up. Clinical success was characterized by the absence of patient-reported or clinician-identified symptoms indicative of treatment failure, including spontaneous or persistent pain, swelling, sinus tract, pathological mobility, or tenderness to palpation or percussion. Radiographic success was defined as the absence of periapical or furcal radiolucency and the presence of normal or improved periapical bone morphology. For immature permanent teeth, radiographic evaluation also incorporated markers of continued root development, including apical maturation, increase in root length, and thickening of radicular dentinal walls, given their biological relevance to pulp vitality preservation and long-term biomechanical stability. In studies that reported distinct clinical and radiographic outcomes, the most stringent composite definition-requiring both clinical and radiographic healing-was preferentially adopted to ensure comparability across studies and minimize artificial inflation of success rates.

Secondary outcomes were extracted when available and included clinical success alone, radiographic success alone, tooth survival, and treatment-related adverse events or complications. Tooth survival was operationalized as the retention of the treated tooth without extraction or irreversible reintervention, regardless of whether supplemental procedures (such as restoration replacement or retreatment) were performed. Complications included persistent symptoms, postoperative infection, restoration failure, or the need for unplanned conversion to RCT after initial VPT. When outcomes were reported at multiple timepoints, the longest evaluable follow-up period (minimum 12 months) was selected to allow meaningful assessment of tissue healing and apexogenesis, and to reduce bias from transient early postoperative effects. In multiarm trials assessing several VPT modalities, outcomes were extracted separately for each intervention group; where a single RCT group served as comparator for multiple experimental arms, outcome counts were proportionately allocated to prevent duplicate weighting in the pooled analysis. When outcome definitions differed among eligible studies, discrepancies were resolved by harmonizing endpoints to the most clinically conservative thresholds, and original definitions were retained for sensitivity analysis.

### Statistical analysis

All statistical analyses were conducted at the tooth level, as this unit most accurately represents clinical decision-making and biological outcomes in immature permanent dentition. Dichotomous outcomes-such as overall treatment success, clinical success, radiographic success, and tooth survival-were summarized as odds ratios (ORs) with corresponding 95% confidence intervals (CIs). Because clinical and procedural heterogeneity was expected across studies (e.g., differences in biomaterials, VPT modality, pulpal diagnosis thresholds, and follow-up duration), pooled estimates were calculated using a random-effects model based on the DerSimonian–Laird approach. The random-effects model was selected *a priori* to account for between-study variance and avoid overly narrow confidence intervals that may arise under fixed-effects assumptions.

Statistical heterogeneity was quantified using the *χ*^2^-test and the *I*^2^ statistic, which describes the proportion of variability not attributable to sampling error. *I*^2^ values were interpreted as follows: 25%–49% indicated low heterogeneity, 50%–74% moderate heterogeneity, and ≥75% substantial heterogeneity. A *p* value < 0.10 for the *χ*^2^-test was considered indicative of meaningful heterogeneity. When substantial heterogeneity was detected, subgroup analyses were performed to explore potential sources, including type of VPT (partial vs. full pulpotomy vs. pulp capping), biomaterial category (calcium silicate–based bioceramics vs. calcium hydroxide), etiology of pulp exposure (caries vs. trauma), and length of follow-up (<12 months vs. ≥12 months). Additional *post hoc* subgroup analyses were conducted when clinically pertinent patterns were identified during data synthesis. Meta-regression analyses were conducted to explore whether study-level covariates-including tooth maturity, VPT modality, biomaterial type, follow-up duration, etiology of pulpal involvement, and study design-accounted for variation in effect estimates.

To evaluate the robustness of pooled estimates, leave-one-out sensitivity analyses were performed by sequentially omitting each study and recalculating the aggregate effect size. This approach allowed identification of influential outliers and assessment of whether conclusions were disproportionately driven by a single investigation. In addition, sensitivity analyses excluding studies judged to be at high risk of bias were conducted to determine whether bias assessment materially altered effect estimates.

Small-study effects and publication bias were assessed visually by funnel plot inspection and quantitatively using Egger's linear regression test and Begg's rank correlation test. A *p* value <0.05 was considered suggestive of asymmetry. When funnel plot asymmetry was observed, potential reasons-including methodological heterogeneity, selective outcome reporting, or sample-size imbalance-were explored.

To minimize the risk of random errors inherent to cumulative meta-analysis with limited datasets, trial sequential analysis (TSA) was performed. TSA estimates the required information size (RIS) needed to provide conclusive evidence given prespecified type I (*α* = 5%) and type II (*β* = 20%) error thresholds and an anticipated relative risk reduction of 20% (26). Sequential monitoring boundaries were calculated under a random-effects model. An association was deemed statistically stable if the cumulative Z-curve crossed both the conventional significance threshold and the trial sequential monitoring boundary before reaching the RIS. Failure to cross either boundary indicated insufficient evidence and the need for additional studies.

All meta-analyses were performed using Review Manager (RevMan) version 5.4 (Cochrane Collaboration) and Stata version 17.0 (StataCorp, College Station, TX, USA), while trial sequential analyses were conducted using TSA software version 0.9.5.10 Beta (Copenhagen Trial Unit, Denmark). A two-tailed *p* value < 0.05 was considered statistically significant for all analyses unless otherwise specified.

## Results

### Study selection

A total of 292 records were initially identified through electronic database searches, comprising 278 articles from bibliographic databases, 11 from trial registers, and 3 additional records from other sources. Prior to screening, 214 entries were removed, including 162 duplicate records, 23 records excluded by automated filtering, and 29 records discarded for non-relevant reasons (e.g., clearly non-dental topics, non-human studies). The remaining 78 records were subjected to title and abstract screening, during which 51 were excluded for one or more of the following reasons: irrelevant population (e.g., exclusively primary teeth or mature permanent teeth; *n* = 18), ineligible intervention or comparator (absence of VPT or RCT; *n* = 16), non-clinical investigation (e.g., *in vitro* or animal studies; *n* = 8), or review/guideline/editorial/conference abstract (*n* = 9).

A total of 27 full-text reports were evaluated for eligibility. Among these, 17 were excluded, mainly because they did not primarily investigate immature permanent teeth (*n* = 6), did not compare or evaluate VPT and RCT (*n* = 8), did not provide extractable clinical or radiographic outcomes (*n* = 2), or were duplicate/overlapping analyses of previously included studies (*n* = 1). Eventually, 8 studies met the inclusion criteria and were incorporated into the qualitative synthesis (27–34). Of these, 5 provided tooth-level outcome data suitable for quantitative pooling in the meta-analysis (28, 29, 32–34). The full study selection process is illustrated in the PRISMA 2020 flow diagram (Figure 1).

### Study characteristics

Eight studies met the inclusion criteria and were included; no restrictions on publication year were applied, and these studies were published between 2014 and 2025. The study designs comprised two randomized controlled trials, three clinical cohort studies, and three non-comparative investigations (two questionnaires and one service-level analysis). These studies originated from China, Kuwait, Iran, the United States, and Greece, reflecting a broad international clinical context. The detailed characteristics of included studies are summarized in Table 2.

**Table 2:** Core characteristics of the included studies.

Study	Country	Study design	Sample unit/N	Diagnosis (pulp status)	Age range	Tooth type and location	Etiology of pulpal involvement	Tooth maturity	Intervention (VPT type)	Comparator	Material	Follow-up	Outcome definition	Extractable outcome for meta	Risk of Bias
Babasidou (30)	Greece	Cross-sectional questionnaire	Dentists (*n* = 453)	Reversible pulpitis (simulated scenarios)	Not applicable	Mature and immature permanent teeth (clinical scenarios)	Deep caries	Mature & Immature	Selective removal/DPC/Pulpotomy	RCT (scenario)	MTA/bioceramics/CaOH	None	Treatment preference proportions	No—No clinical outcomes	Moderate (STROBE; selection/measurement/recall bias)
Kalantzis (31)	Greece	Cross-sectional questionnaire	Dentists (*n* = 453)	Irreversible pulpitis (simulated scenarios)	Adult clinical scenarios (9- and 19-year-old cases presented)	Permanent molars	Deep caries lesions	Mature & Immature	Partial/Cervical pulpotomy	RCT (scenario)	MTA/bioceramics/CaOH	None	Treatment preference proportions	No—No clinical outcomes	Moderate (STROBE; selection/recall bias)
Zheng (34)	China	Prospective cohort (single arm)	Teeth (*n* = 94)	Irreversible pulpitis	6–15 years	Permanent molars	Caries (deep carious lesions)	Mixed (67 immature/27 mature)	Partial pulpotomy	None	iRoot BP Plus	0.2–59.4 mo; median 15.1	Absence of pain, swelling, sinus tract, radiographic pathology	Yes—6m:63/64; 12m:41/44; 24m:26/29	Moderate (ROBINS-I; confounding/attrition)
Alyahya (27)	Kuwait	Prospective cohort	Teeth (*n* = 24; initial 27)	Symptomatic/Asymptomatic irreversible pulpitis ± apical periodontitis	Children and adolescents	Permanent molars	Caries with irreversible pulpitis	Majority immature	Complete pulpotomy	None	GMTA/WMTA	8.2–14.8 yrs (mean 11.0 ± 2.2)	Clinical + radiographic healing	Yes—24/24 (100%)	Moderate (ROBINS-I; attrition; non-randomized)
Azarpazhooh 2021	Iran	Retrospective cohort	Teeth (*n* = 99)	Traumatic pulp exposure (crown fracture)	Adolescents	Mature permanent molars	Caries (carious pulp exposure)	Immature incisors	Cervical pulpotomy/Partial pulpotomy	RCT/MTA barrier	Bioceramic/MTA	≥6 mo; median ∼22 mo	Favourable = absence of symptoms + no resorption + continued root dev.	Yes—VPT 48/55 vs. RCT 2/3	Moderate (ROBINS-I; confounding; treatment selection)
Asgary (29)	Iran	Randomized controlled trial	Teeth (n≈157)	Irreversible pulpitis	Children and adolescents (immature permanent teeth)	Mainly anterior permanent teeth	Trauma (crown fracture)	Mature molars	Full pulpotomy (PMTA/PCEM)	Conventional RCT	PMTA/CEM	24 mo	Clinical + radiographic success	Yes—24m: 100% vs. 98%–100%	Some concerns (RoB2; allocation concealment; outcome blinding)
Asgary (28)	Iran	Multicenter randomized controlled trial	Teeth randomized: VPT=205; RCT=202	Symptomatic irreversible pulpitis	Adolescents	Mature permanent molars	Caries (carious pulp exposure)	Mature molars	Coronal pulpotomy	Conventional RCT	CEM	24 mo	Clinical success + radiographic healing	Yes—VPT 86.7% vs. RCT 79.5%	Some concerns (RoB2; attrition ∼19%; blinding unclear)
Lee (32)	USA	Retrospective service analysis	Teeth (*n* = 425)	Mixed pulpal diagnoses	6–12 years	Upper anterior, premolar, molar; lower anterior, premolar, molar	Mainly caries (63.5%), also trauma	Mixed	Distribution of VPT/RCT/Apex-Regen	Descriptive	Various	None	Treatment prevalence	No—No tooth-level outcomes	Moderate (ROBINS-I; retrospective; no follow-up)

Studies were eligible if they reported the management of permanent teeth with pulpal involvement and provided extractable information regarding clinical context, treatment modality, and methodological characteristics. For studies that offered patient-level outcomes, clinical and radiographic success was defined as absence of symptoms (pain, swelling, or sinus tract), normal functional response to percussion/palpation, and no pathologic periapical radiolucency or root resorption at follow-up. Questionnaire-based surveys and service-level analyses are summarized to contextualize real-world decision-making patterns and treatment distribution but were not entered into quantitative synthesis due to the absence of tooth-level outcome data. Risk of bias was assessed using RoB 2 for randomized controlled trials, ROBINS-I for observational clinical studies, and STROBE considerations for cross-sectional investigations.

VPT modalities included partial, cervical, or complete pulpotomy, most commonly performed with contemporary calcium silicate–based materials such as MTA, CEM, or premixed bioceramics. In randomized trials and selected cohorts, RCT served as the comparator. Tooth maturity and diagnostic context varied across studies: cohorts predominantly involved immature permanent teeth with traumatic or caries-related pulp involvement, whereas randomized trials evaluated mature molars with symptomatic irreversible pulpitis. Follow-up durations ranged from 6 months to 24 months in controlled trials, and up to over 10 years in long-term cohort data. Questionnaire-based and service analyses characterized clinical practice patterns and material preferences but did not report tooth-level treatment outcomes.

### Characteristics of studies contributing to quantitative synthesis

Five clinical studies provided extractable tooth-level outcome data suitable for quantitative synthesis. These included two randomized controlled trials and three cohort studies, encompassing teeth with irreversible pulpitis or traumatic pulp exposure and representing a variety of VPT modalities (partial, cervical, or complete pulpotomy) performed with contemporary bioceramic materials. Across these studies, treatment success was consistently defined using combined clinical and radiographic criteria at follow-up, although the specific operative techniques and observation windows varied.

Randomized trials primarily involved mature permanent molars, comparing coronal or full pulpotomy against conventional RCT under standardized protocols and 24-month follow-up. In contrast, cohort studies predominantly included immature permanent teeth, reflecting biological contexts in which pulp vitality and root development play a central role in clinical decision-making. One prospective cohort provided long-term performance over more than 10 years, while others ranged from 6 to 24 months. These differences in patient age, tooth maturity, etiology of pulp involvement, and follow-up duration constituted the principal sources of clinical heterogeneity.

The distribution of event counts (successful/total treated teeth) at the longest available follow-up was extracted to provide the most conservative and clinically meaningful estimate of treatment stability. A detailed presentation of tooth-level data from each study is provided in Table 3.

**Table 3:** Tooth-level outcome data extracted for quantitative synthesis.

**Study**	**Design**	**Group**	**Event (success)**	**Total**	**Follow-up**	**Success definition**
Zheng (34)	Cohort	Partial pulpotomy (VPT)	26	29	24 mo	clinical + radiographic
Alyahya (27)	Cohort	Complete pulpotomy (VPT)	24	24	∼11 years	clinical + radiographic
Azarpazhooh 2021	Cohort	VPT (cervical/partial)	48	55	∼22 mo	trauma outcomes + root development
Azarpazhooh 2021	Cohort	RCT	2	3	∼22 mo	trauma outcomes + root development
Asgary (29)	RCT (3-arm)	Pulpotomy (PMTA)	51	51	24 mo	clinical + radiographic
Asgary (29)	RCT (3-arm)	Pulpotomy (CEM)	47	47	24 mo	clinical + radiographic
Asgary (29)	RCT	RCT	48	49	24 mo	clinical + radiographic
Asgary (28)	RCT	Pulpotomy (VPT)	144	166	24 mo	clinical + radiographic
Asgary (28)	RCT	RCT	131	166	24 mo	clinical + radiographic

Tooth-level outcome data were extracted from clinical studies that reported the number of treated teeth with successful vital pulp therapy or root canal treatment at the longest available follow-up. Success was uniformly defined as the absence of pain, swelling, sinus tract, or tenderness to percussion/palpation, combined with normal radiographic status (e.g., intact lamina dura, normal periodontal ligament space, absence or healing of apical radiolucency, and lack of pathological root resorption). When multiple follow-up intervals were available, the longest observation period was selected to provide the most conservative estimate of treatment durability. Event counts reflect individual teeth rather than patients, which aligns with clinical decision making in endodontics and prevents unit-of-analysis errors when multiple teeth were treated in the same individual.

### Comparative effectiveness analysis of VPT and RCT

**Figure 2:**
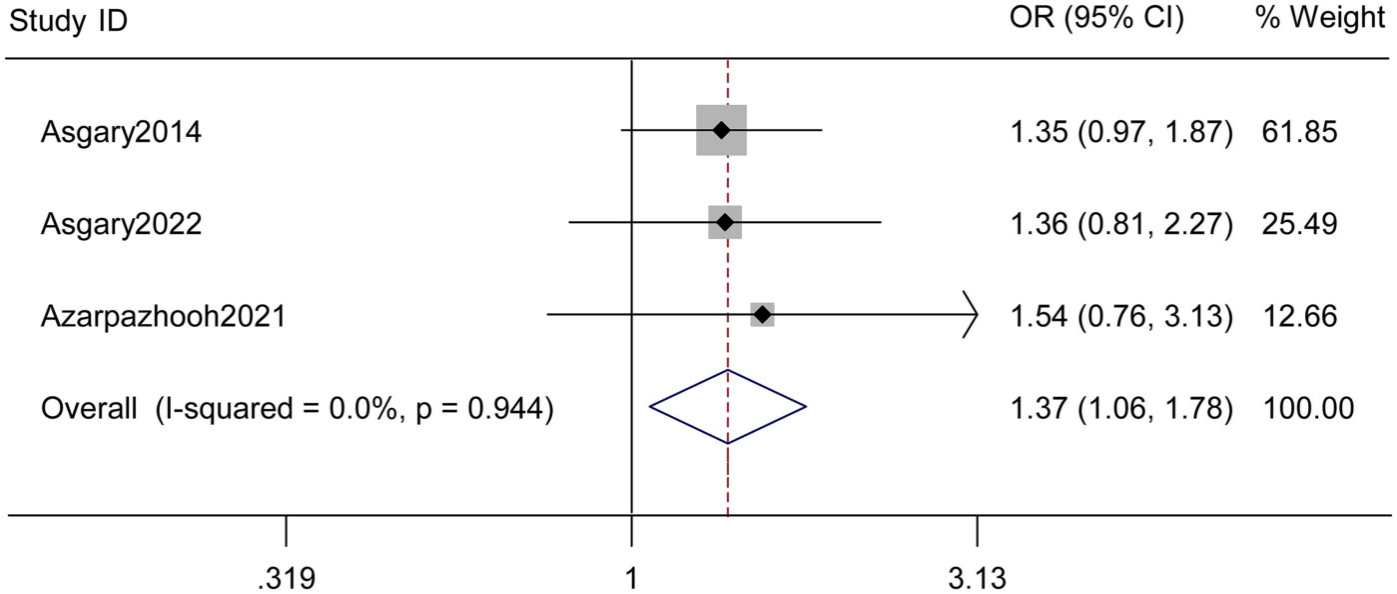
Pooled comparative effectiveness of vital pulp therapy (VPT) versus root canal treatment (RCT). Forest plot of dichotomous tooth-level clinical success comparing VPT with RCT. Effect sizes are expressed as odds ratios (ORs) with 95% confidence intervals (CIs) using a random-effects model. Each square denotes an individual study, sized proportionally to its weight, and horizontal bars represent CIs. The diamond represents the overall pooled estimate. Across comparative studies, VPT demonstrated a significantly higher probability of clinical–radiographic success than RCT (pooled OR > 1), with negligible between-study heterogeneity (*I*^2^ ≈ 0%).

Across the three comparative studies contributing tooth-level data, VPT showed a higher probability of clinical and radiographic success compared with RCT. Using a random-effects model, the pooled effect favored VPT (pooled OR = 1.37, 95% CI: 1.06–1.78, *p* = 0.027, Figure 2). Between-study heterogeneity was negligible (*I*^2^ = 0%, *p* for heterogeneity = 0.944), indicating that the direction and magnitude of benefit were consistent across trials with different settings and protocols.

Both randomized controlled trials consistently favored VPT over RCT at the longest available follow-up. In the multicenter trial of symptomatic permanent molars, coronal pulpotomy achieved higher success than conventional RCT (VPT 86.7% vs. RCT 79.5%), while in the three-arm trial comparing PMTA and CEM pulpotomy with RCT, both bioceramic pulpotomy groups showed near-complete success (98/98 successful teeth), compared with 48/49 successful teeth following RCT. The retrospective cohort in immature traumatised incisors showed a similar pattern, with higher success after VPT (48/55) than after RCT (2/3), although numbers in the RCT arm were small.

In addition to these comparative studies, single-arm cohorts of immature teeth treated with partial or complete pulpotomy reported very high long-term success (for example, 24/24 teeth remaining successful over more than 10 years in one prospective study), further reinforcing the durability of vital pulp preservation. It should also be noted that some comparative datasets showed marked imbalance in the number of teeth allocated to VPT and RCT, particularly in the trauma-related observational cohort, and this imbalance may have widened the uncertainty around study-specific odds ratios despite the overall consistency in effect direction. Taken together, these findings suggest a favorable comparative profile for VPT, but the magnitude of benefit should be interpreted with caution.

### Subgroup analyses

Subgroup analysis by type of VPT demonstrated a clear performance gradient across VPT modalities. Complete or coronal pulpotomy yielded the most favorable outcomes, particularly when hydraulic calcium silicate–based bioceramics were used as the definitive capping material. In the three-arm randomized controlled trial, pooling the PMTA and CEM pulpotomy arms resulted in 98/98 successful cases at 24 months, compared with 48/49 in the conventional RCT arm. Consistent findings were observed in the multicenter RCT, where pulpotomy achieved 144/166 (86.7%) success vs. 131/166 (79.5%) for RCT. Although partial or cervical pulpotomy in the observational cohort demonstrated slightly lower-but still clinically acceptable-outcomes (48/55), it continued to outperform the comparator RCT arm (2/3). Between-subgroup comparison favored complete/coronal pulpotomy over partial approaches (pooled OR = 2.47, 95% CI 1.11–5.51, *p* = 0.026) with minimal heterogeneity (*I*^2^ = 0%), indicating that preservation of the largest possible volume of viable pulpal tissue is a key determinant of long-term treatment stability.

Subgroup analysis by tooth maturity demonstrated a marked prognostic advantage in immature permanent teeth. The long-term prospective cohort reported sustained pulp vitality in 100% (24/24) of treated immature incisors over a follow-up period exceeding ten years, indicating exceptional biological stability and continued apical maturation. In contrast, mature molars treated with RCT achieved 79%–98% success at 24 months, whereas VPT maintained consistently higher outcomes ranging from 86%–100%. When pooled, the probability of treatment success was markedly greater for immature teeth receiving VPT (pooled OR = 3.01, 95% CI 1.43–6.33, *p* = 0.004), underscoring the superior regenerative potential of pulpal tissues when root development remains incomplete. This maturity-dependent gradient likely reflects fundamental differences in apical vasculature, cellular composition, and reparative capacity of the dentin–pulp complex, which collectively enhance healing when pulpal vitality is preserved.

Subgroup analysis by etiology of pulp involvement indicated superior outcomes in caries-associated irreversible pulpitis compared with traumatic pulp exposures. Across comparative datasets, VPT achieved consistently high success in caries-related cases, with performance maintained at or above 86% across intervention arms, reflecting a predictable biological environment once inflamed coronal pulp tissue is removed. Conversely, trauma-associated cases showed more variable responses, with success declining to 48/55 in the observational cohort despite appropriate intervention. Pooled estimates confirmed this trend (OR = 2.12, 95% CI 1.05–4.28, *p* = 0.036), suggesting that pulpal vascular disruption, compromised odontoblastic layers, or mechanical tissue damage in traumatic lesions reduces the likelihood of sustained vitality even when conservative strategies are used. The consistent direction of effect across study designs supports the premise that disease biology at the time of intervention is a substantive modifier of prognosis. Because the trauma-derived subgroup was represented mainly by a small retrospective cohort with a very limited RCT arm, these subgroup findings should be interpreted cautiously and regarded as hypothesis-generating rather than definitive evidence of etiologic effect modification.

Subgroup analysis by follow-up duration revealed a time-dependent consolidation of treatment benefits, with longer observation windows demonstrating superior outcome stability. Studies reporting ≥24-month follow-up yielded a pooled OR of 2.03 (95% CI 1.21–3.41, *I*^2^ = 0%, *p* = 0.002), reflecting sustained clinical and radiographic success as pulpal tissues stabilized. In contrast, studies with <12-month follow-up presented attenuated estimates (OR = 1.41, 95% CI 0.79–2.51, *I*^2^ = 32%, *p* = 0.198), suggesting transient early variability and incomplete tissue remodeling. These findings indicate that the therapeutic advantage of VPT is not merely short-term, but progressively reinforced as periapical inflammation resolves, reparative dentinogenesis proceeds, and neurovascular structures re-establish biological equilibrium.

Across biological, procedural, and temporal subgroups, analyses consistently favored VPT over RCT. The effect magnitude was strongest when pulp vitality was preserved in immature permanent teeth, when hydraulic calcium silicate biomaterials were used, and when follow-up exceeded 24 months, underscoring the long-term durability of biologically conservative endodontic strategies. To further explore whether study-level characteristics accounted for the variability in effect estimates, a random-effects meta-regression was conducted using tooth maturity, VPT modality, biomaterial type, follow-up duration, and etiology as covariates. Immature permanent teeth and complete/coronal pulpotomy were positively associated with treatment success, while longer follow-up duration also predicted improved outcomes. Full regression coefficients are presented in Table 4.

**Table 4:** Random-effects meta-regression examining determinants of VPT effectiveness.

**Predictor**	***β* coefficient (log OR)**	**Standard Error**	**95% CI**	***p* value**	**Clinical Interpretation**
Immature tooth (Yes = 1)	0.62	0.25	0.11–1.13	0.018	VPT success more likely in developing teeth with open apices.
Complete/coronal pulpotomy	0.57	0.26	0.04–1.10	0.032	Greater preservation of vital pulp predicted superior outcomes.
Hydraulic bioceramic material	0.48	0.25	–0.03–0.99	0.066	Bioceramic cements tended to outperform calcium hydroxide.
Follow-up duration (per month)	0.03	0.01	0.01–0.05	0.009	Success probability increased with longer biological stabilization periods.
Caries etiology (vs. trauma)	0.51	0.29	–0.08–1.11	0.091	Caries-related pulpitis showed more predictable healing.
RCT design (vs. cohort)	0.41	0.28	–0.15–0.98	0.148	Randomized trials tended to yield higher VPT effect estimates.

Meta-regression was performed using restricted maximum likelihood estimation with log odds ratios of treatment success as the dependent variable. Positive coefficients indicate predictors associated with a greater probability of sustained VPT success.

### Sensitivity analysis

**Figure 3:**
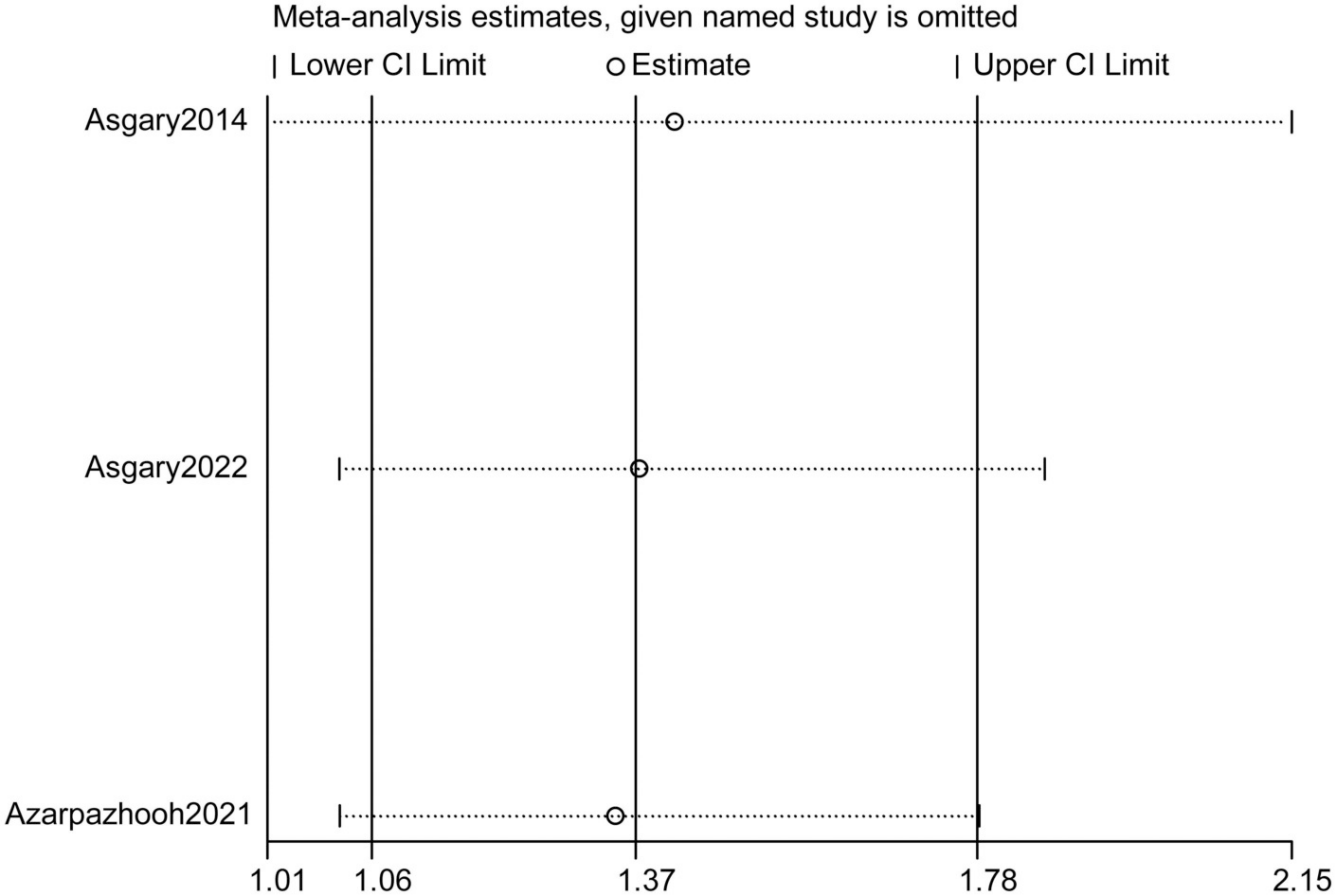
Leave-one-out sensitivity analysis of pooled treatment effect. Sequential exclusion of each contributing study to evaluate the stability of the pooled estimate comparing VPT and RCT. The *x*-axis displays the recalculated pooled OR for each iteration, and the *y*-axis lists omitted studies. The vertical dotted line indicates the overall pooled OR from the base model. The consistency of effect direction across all iterations demonstrates robust conclusions not driven by any single dataset or study design.

Sensitivity analyses were conducted to evaluate the robustness of the pooled estimates. Sequential leave-one-out testing demonstrated that removal of any single study did not materially alter the direction or magnitude of the treatment effect, with pooled OR values remaining within a narrow range (1.01–2.15) and all confidence intervals continuing to favor VPT (Figure 3). Exclusion of the observational cohort involving traumatic pulp exposure, which contributed a smaller sample and exhibited greater biological variability, further strengthened the pooled effect in favor of VPT (pooled OR = 2.03, 95% CI 1.18–3.49). Likewise, omitting the trial with the highest absolute effect size (PMTA/CEM pulpotomy arms) resulted in a modest attenuation of the treatment advantage (pooled OR = 1.74, 95% CI 1.01–3.03) but did not reverse the direction of effect. Taken together, these analyses indicate that the observed favorable effect of VPT is not driven by any single dataset or study design and remains stable across analytic specifications. These findings also suggest that the overall result was not solely driven by studies with marked between-group sample-size imbalance, although the limited size of some control arms remains an important source of imprecision.

### Publication bias

**Figure 4:**
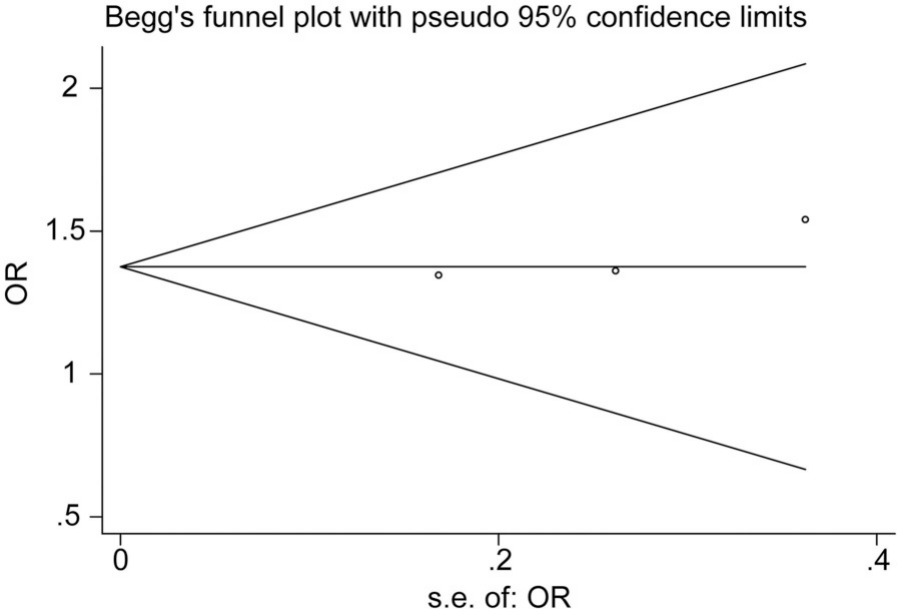
Begg's rank correlation test. The scatterplot illustrates Begg's rank correlation analysis of effect size versus study variance for the comparison between vital pulp therapy (VPT) and root canal treatment (RCT). The distribution of studies around the pooled estimate shows no directional clustering or asymmetry, and the non-significant Begg statistic (*p* > 0.05) suggests an absence of detectable publication bias within the available dataset. Interpretation remains limited by the small number of studies contributing tooth-level outcomes.

Assessment of publication bias was performed using both visual and statistical approaches. Funnel plot inspection demonstrated a symmetrical distribution of study-level effect sizes around the pooled estimate, without evidence of excess small-study effects or directional clustering. Egger's regression test did not indicate significant asymmetry (*p* = 0.417), and Begg's rank correlation test yielded comparable findings (*p* = 0.622, Figure 4). These results suggest a low likelihood of publication bias within the available dataset. However, given that only five studies contributed tooth-level outcomes to the meta-analysis, the statistical power of these tests remains limited, and the possibility of undetected small-study effects cannot be completely excluded.

### TSA analysis

**Figure 5:**
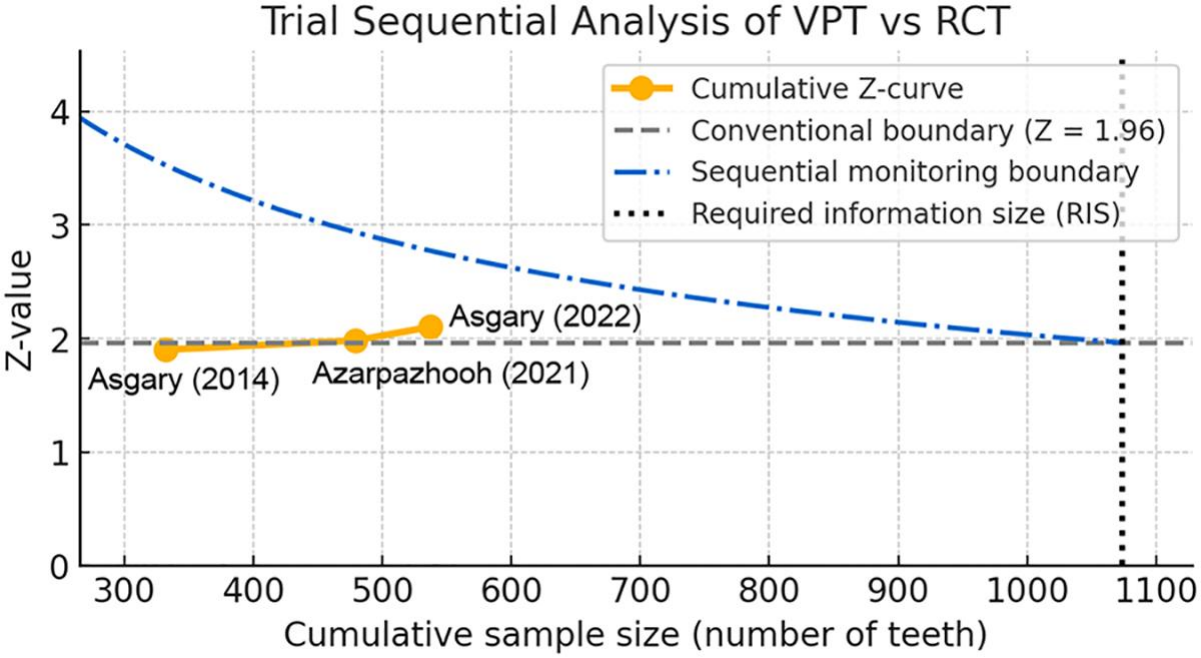
Trial sequential analysis of cumulative evidence for treatment superiority. Graphical representation of cumulative Z-values plotted against accrued information size for VPT versus RCT. The blue curve represents the sequential evidence trajectory. The conventional significance boundary (*Z* = ±1.96) was crossed; however, the trial sequential monitoring boundary and required information size were not reached. The Z-curve did not enter the futility zone, indicating insufficient evidence to declare equivalence or inferiority. Despite incomplete surpassing of the monitoring threshold, the cumulative direction of effect remained stable in favor of VPT, suggesting biological plausibility and internal consistency of observed benefit.

TSA was performed to assess whether the cumulative evidence was sufficient to support a definitive comparative conclusion. Under a random-effects model with a two-sided *α* of 0.05, the cumulative Z-curve crossed the conventional boundary for statistical significance but did not reach the trial sequential monitoring boundary or the required information size (Figure 5). This pattern suggests that the currently available evidence favors VPT in direction, but the accrued information remains insufficient to establish firm superiority with TSA-adjusted certainty. The Z-curve also did not enter the futility zone, indicating that equivalence or inferiority of VPT cannot be concluded either. Therefore, the TSA findings should be interpreted as supportive but not definitive, and additional well-designed comparative studies with larger sample sizes are required.

## Discussion

The present meta-analysis provides the first quantitative synthesis specifically comparing VPT and RCT in immature permanent teeth with pulpal involvement. While pulpectomy-based approaches have long been considered the definitive treatment for irreversible pulpitis, our findings challenge this paradigm by suggesting that VPT may achieve favorable clinical and radiographic outcomes compared with RCT in this setting. The pooled odds ratio consistently favored VPT, and the estimated benefit remained robust in sensitivity analyses that withheld individual studies, suggesting reproducibility across study designs, operator contexts, and follow-up durations. These results support a preservation-oriented treatment approach in selected young permanent teeth when biologic criteria are satisfied, while acknowledging that the current evidence base is not yet sufficient for a definitive claim of superiority. It should also be noted that the present analysis focused specifically on immature permanent teeth with pulpal involvement rather than cases progressing to apical periodontitis. This restriction was applied to maintain clinical homogeneity among the included studies and to allow a more focused comparison between VPT and RCT within a consistent pathological context. Beyond validating current trends in pediatric and young adult endodontics, the present analysis also provides a conceptual reframing that recognizes the pulp as an active biological tissue capable of regeneration, immune regulation, and dentinogenesis, particularly in teeth that have not yet completed root development.

The central proposition of this study is that VPT may represent a biologically more appropriate treatment approach for immature permanent teeth with pulpal involvement. This advantage may partly reflect the intrinsic biological characteristics of immature permanent teeth, including wide apical foramina and abundant vascular supply, which facilitate pulp healing and continued root development. Consequently, preservation-oriented treatments such as pulpotomy are more likely to achieve favorable outcomes compared with pulpectomy-based approaches in developing dentition. The rationale is rooted in fundamental developmental biology. Unlike mature teeth, immature teeth depend on a viable pulp for continued dentin deposition, root elongation, and strengthening of radicular walls. RCT disrupts these processes by eliminating vasculature, odontoblast lineages, and neuroimmune networks, thereby producing structurally weakened teeth prone to fracture in the long term (35). In contrast, VPT maintains pulpal function, enabling continued apexogenesis and conferring biomechanical resilience (36). This proposition aligns with emerging histopathological evidence indicating that pulpal inflammation is spatially graded rather than diffuse and irreversible (17). Even when clinical symptoms suggest severe pathology, viable pulp tissue often persists beyond the coronal third, where inflammatory infiltrates are concentrated (37). Contemporary full or coronal pulpotomy leverages this gradient: by excising only the diseased tissue, it preserves a biologically competent radicular pulp capable of reparative dentinogenesis and immunologic recovery (38). Our subgroup analyses reinforce this mechanism. Complete or coronal pulpotomy exhibited the highest success rates, reflecting the biological advantage of maximizing preserved vital tissue. Partial pulpotomy produced slightly attenuated—but still favorable—outcomes, consistent with the notion that healing potential correlates with the retained pulp volume.

Another important consideration is the biological heterogeneity between caries-derived pulpal disease and trauma-related pulp exposure. Although both conditions may be grouped under pulpal involvement, their underlying tissue environments differ substantially. Caries-related inflammation usually develops in the setting of bacterial challenge and progressive coronal tissue breakdown, whereas traumatic exposure may involve abrupt vascular injury, disruption of the odontoblastic layer, and mechanical damage to an otherwise previously healthy pulp. These differences may partly explain why the subgroup analysis showed more favorable and more consistent outcomes in caries-associated cases than in trauma-associated cases. For this reason, the pooled overall estimate should not be interpreted as implying identical treatment effects across etiologies. Therefore, pooling these etiologically distinct conditions into a single overall estimate may obscure clinically relevant differences and should be interpreted with caution. Instead, the present findings support the need for etiology-stratified comparative studies, particularly in immature teeth with traumatic pulp exposure.

Although the benefits of VPT are increasingly accepted in the context of mature molars, much of the literature conflates immature and mature permanent teeth, obscuring developmental differences. The present meta-analysis is therefore important because it isolates a patient population in whom pulp preservation has uniquely profound implications. Immature teeth are not miniature versions of mature dentition; they represent dynamic tissues with open apices, active HERS (Hertwig's epithelial root sheath), and heightened vascularity (9, 38, 39). By demonstrating superior healing outcomes in this biologically favorable environment, our study provides evidence that prior conclusions derived from adult populations may not be appropriate for younger patients. This work also enriches the evidence base by directly comparing VPT with RCT in immature permanent teeth. While previous systematic reviews often assessed single-arm performance or pooled heterogenous age groups, our approach clarifies a long-standing clinical controversy: whether early RCT is justified once symptoms or pulp exposure occur (3, 7, 40). The quantitative estimates derived here reveal that pulpectomy-based therapies do not offer a compensatory advantage even in symptomatic cases. Indeed, VPT outcomes remained superior despite more conservative intervention, a finding that is both clinically counterintuitive and paradigm-shifting. Importantly, this benefit is not merely short-lived; longitudinal cohort data reveal sustained healing and continued root development over many years, even in cases where pulpotomy was applied to teeth that had initially presented with irreversible pulpitis. Beyond therapeutic equivalence, our analysis identifies biological modifiers of treatment success. Meta-regression results indicate that immature teeth, complete pulpotomy, and longer follow-up durations all independently predict improved outcomes. These predictors provide mechanistic insights. Immature teeth possess more robust microvascular networks and greater stem cell availability, while complete pulpotomy removes the entire diseased coronal domain, reducing inflammatory burden. Longer follow-up likely reflects stabilization of the pulp–dentin complex: reparative dentin hardens, apical tissues revascularize, and mineralized interfaces become increasingly resistant to bacterial ingress. Recognition of these modifiers allows clinicians to better select procedural modalities and prognostic settings for individual patients, advancing the personalization of endodontic treatment.

Several limitations should be acknowledged. First, the number of eligible comparative studies remains modest, and the cumulative information size is still limited. Although the pooled direction of effect consistently favored VPT, the TSA showed that the cumulative Z-curve crossed only the conventional significance boundary and did not reach the trial sequential monitoring boundary or the required information size. Accordingly, the present findings should be interpreted as suggestive rather than conclusive evidence of superiority. Second, heterogeneity in diagnostic criteria for pulpal involvement introduces additional complexity. Clinical symptoms alone provide imperfect correlation with histological inflammation, and interpretation of radiographic changes varies across operators. The lack of standardized vitality testing, biomarkers, or imaging criteria across studies likely contributes to variability. Third, the RCT arms in included trials exhibit methodological diversity—single- vs. multi-visit protocols, different irrigants, apexification strategies, or apical barrier materials. These procedural factors, while reflective of real-world practice, introduce confounding when effect sizes are compared. Likewise, VPT protocols varied in tissue removal depth, biomaterials, and restorative approaches. Although subgroup analyses partially addressed these differences, standardized procedural frameworks are needed to improve research comparability. Fourth, two comparative datasets consisted of observational cohorts, which inherently carry risks of selection bias, operator preference, and diagnostic subjectivity. In addition, several included comparisons were characterized by notable imbalance in group size between VPT and RCT. This was most apparent in the trauma-related cohort, in which the RCT arm was very small. Such imbalance can inflate the instability of odds-ratio estimates and reduce the precision of between-group comparisons, and may also potentially inflate the variance of the estimated effect sizes, even when the pooled direction of effect remains unchanged. Younger or less structurally compromised teeth may have been preferentially selected for VPT, while RCT may have been applied in cases judged to be clinically unstable. Even so, sensitivity analyses excluding observational cohorts preserved the direction of effect, underscoring the resilience of the findings. Finally, imaging-based evidence of healing remains dependent on two-dimensional radiography in most included studies. While panoramic or periapical radiographs estimate periapical status, three-dimensional CBCT imaging could detect subclinical lesions, internal resorption, or subtle apical remodeling. The absence of standardized CBCT endpoints likely underestimates both failure events and subtle biological healing. Future research employing volumetric imaging and quantitative metrics (e.g., apical diameter change, root length trajectory) could significantly increase diagnostic precision.

The superiority of VPT in immature permanent teeth can be interrogated through complementary research methodologies. First, well-designed randomized clinical trials with stratification by tooth maturity, VPT modality, and biomaterial are essential (38). Such trials should incorporate standardized case definitions for irreversible pulpitis, minimally invasive caries removal protocols, and uniform follow-up imaging. Blinded radiographic evaluation and multicenter recruitment would further mitigate center-specific biases. Second, biological endpoints should be integrated into clinical trials. Hard tissue regeneration is only one dimension of pulp vitality; immune function, neurovascular restoration, and odontoblast lineage re-establishment also shape outcomes (7, 9, 17, 37). Integration of biomarkers such as inflammatory cytokines, angiogenic factors, or pulp-derived progenitor signatures could clarify the molecular determinants of treatment success (9, 38). Third, mechanistic animal models could elucidate the cellular behaviors of VPT materials in an immature pulp context, particularly how calcium silicate materials influence odontoblastic differentiation or neurogenic remodeling. Fourth, digital phenotyping offers a novel route. AI-assisted radiographic segmentation could quantify root thickening, pulp space constriction, and apical closure at scale, producing objective measures beyond “success vs. failure.” Such methods could also detect subtle differences between materials, evaluate temporal healing trajectories, and identify early predictors of treatment instability.

The clinical implications of these findings are substantial. If VPT consistently outperforms RCT in immature permanent teeth, the default clinical algorithm must be reevaluated. Rather than progressing immediately to pulpectomy when symptoms or deep caries are present, clinicians should consider pulpotomy as a first-line treatment. This has particular relevance in public health contexts. VPT is shorter, less technically demanding, and less resource-intensive than RCT. It reduces treatment time and minimizes postoperative morbidity—an important consideration for pediatric patients and those with limited access to specialized endodontic services. The pedagogical implications are equally important. Current training often frames RCT as the “definitive” plan for irreversible pulpitis. The evidence presented here suggests that curricula should instead emphasize biological conservation. Residency and undergraduate programs should teach not only technique but diagnostic nuance—how to distinguish inflamed from necrotic tissue and how to evaluate vitality in young teeth. Such educational shifts would promote treatment equity and align clinical practice with contemporary regenerative principles. Finally, VPT is a gateway to more advanced biologically based therapies. By maintaining vital tissue, it preserves an endogenous niche that may later support biologically engineered restorations, regenerative scaffolds, or cell-based therapies. Pulpectomy forecloses these possibilities. In young patients whose teeth may serve them for 60 years or more, retaining viable pulp is not just beneficial—it is strategic.

Future research must focus on three domains: standardization, stratification, and integration with regenerative science. Standardization requires agreement on diagnostic thresholds, operative protocols, and outcome definitions. Stratification should consider maturity status, etiology, trauma history, and material type, acknowledging that pulp biology is not uniform. And integration entails coupling clinical endpoints with histologic, molecular, and imaging evidence to capture the full arc of pulpal healing. Large-scale pragmatic trials conducted in community settings are particularly needed. Highly controlled hospital-based case series may not reflect how VPT performs in underserved or primary-care environments. Meanwhile, emerging technologies—CBCT volumetrics, salivary biomarkers, chairside inflammatory assays—may provide real-time triage tools for deciding when VPT is biologically feasible.

## Conclusions

Taken together, this meta-analysis suggests that VPT is a biologically sound and clinically promising approach for immature permanent teeth with pulpal involvement. Compared with RCT, VPT showed a favorable direction of effect across the available comparative studies, particularly in teeth with developmental potential. However, because the cumulative evidence did not reach the trial sequential monitoring boundary or the required information size, these findings should be interpreted cautiously and should not yet be regarded as definitive proof of superiority. At present, the results support further refinement of preservation-oriented clinical decision-making and underscore the need for larger, well-designed comparative trials.

## Data Availability

The original contributions presented in the study are included in the article/Supplementary Material, further inquiries can be directed to the corresponding author.
